# Blockade of the chemokine receptor, CCR5, reduces the growth of orthotopically injected colon cancer cells via limiting cancerassociated fibroblast accumulation

**DOI:** 10.18632/oncotarget.10227

**Published:** 2016-06-22

**Authors:** Yamato Tanabe, Soichiro Sasaki, Naofumi Mukaida, Tomohisa Baba

**Affiliations:** ^1^ Division of Molecular Bioregulation, Cancer Research Institute, Kanazawa University, Kanazawa, Japan

**Keywords:** colorectal cancer, CCL3, CCR5, cancer-associated fibroblast, maraviroc

## Abstract

We previously demonstrated that cancer-associated fibroblasts (CAFs) accumulate at tumor sites through the interaction between a chemokine, CCL3, and its receptor, CCR5, in the late phase of colitis-associated colon carcinogenesis. Here we examined the effect of a CCR5 antagonist, maraviroc, on tumor growth arising from the orthotopic injection of mouse or human colon cancer cell lines into the cecal wall by focusing on CAFs. Orthotopic injection of either cell line caused tumor formation together with leukocyte infiltration and fibroblast accumulation. Concomitant oral administration of maraviroc reduced tumor formation with few effects on leukocyte infiltration. In contrast, maraviroc reduced the intratumor number of α-smooth muscle actin-positive fibroblasts, which express epidermal growth factor, a crucial growth factor for colon cancer cell growth. These observations suggest that maraviroc or other CCR5 antagonists might act as novel anti-CRC drugs to dampen CAFs, an essential cell component for tumor progression.

## INTRODUCTION

Colorectal cancer (CRC) is the third most commonly diagnosed cancer (1.36 million cases) and the fourth highest cause of cancer deaths (0.7 million) worldwide [1]. Approximately three quarters of patients newly diagnosed with CRC exhibit localized disease that is amenable to curative surgical resection, whereas the remaining patients present with unresectable metastatic disease [2]. Furthermore, a substantial proportion of patients experience disease recurrence or metastatic disease after surgical resection. The prognosis of CRC patients with metastasis and/or recurrence has been improved by the use of various types of chemotherapeutic drugs including 5-fluorouracil, leucovorin, irinotecan, and oxaliplatin [2, 3]. However, the median overall survival (OS) still remains about 2 years [2]. Thus, an additional treatment modality is required for advanced CRC.

Accumulating evidence indicates the crucial involvement of inflammation in cancer progression and metastasis [3]. Inflammation is pathologically characterized by the presence of inflammatory cell infiltration, fibrosis ensuing from fibroblast accumulation, and angiogenesis [4]. Angiogenesis is presumed to promote cancer progression and metastasis by supplying cancer cells with oxygen and nutrients, which are indispensable for cancer cell growth [5]. As a consequence, an anti-vascular endothelial growth factor (VEGF) antibody is used to supplement cytotoxic chemotherapy for CRC [6]. However, the antibody can prolong OS of patients with metastatic CRC by only a few months [2]; therefore, additional targets need to be identified for the establishment of a therapy for advanced CRC.

Fibroblasts present in tumor tissues, designated as cancer-associated fibroblasts (CAFs), can provide cancer cells with scaffolds and growth factors to promote cancer cell growth [7]. For example, the contribution of CAF-derived growth factors has been documented in a mouse colitis-associated CRC model [8, 9]. We further revealed that in this model, a chemokine, CCL3, induces the accumulation of fibroblasts that express CCR5, a specific receptor for CCL3 [10]. Additionally, genetic deletion of the *Ccr5* gene or *in vivo* overexpression of a dominant negative form of the CCR5 ligand, CCL5, attenuated tumor formation in this model together with reduced fibroblast accumulation. Furthermore, we observed that CCR5-deficient mice exhibited slower tumor growth than wild-type mice when a syngeneic mouse CRC cell line was injected either subcutaneously or orthotopically into the cecal wall [10]. Therefore, in this study we investigated the potential of a clinically utilized CCR5 antagonist, maraviroc, for the treatment of CRC in mouse pre-clinical models.

## RESULTS

### Orthotopic injection of a murine CRC cell line forms tumors in association with CCR5-expressing fibroblasts and CCL3-expressing granulocytes and macrophages

**Figure 1:**
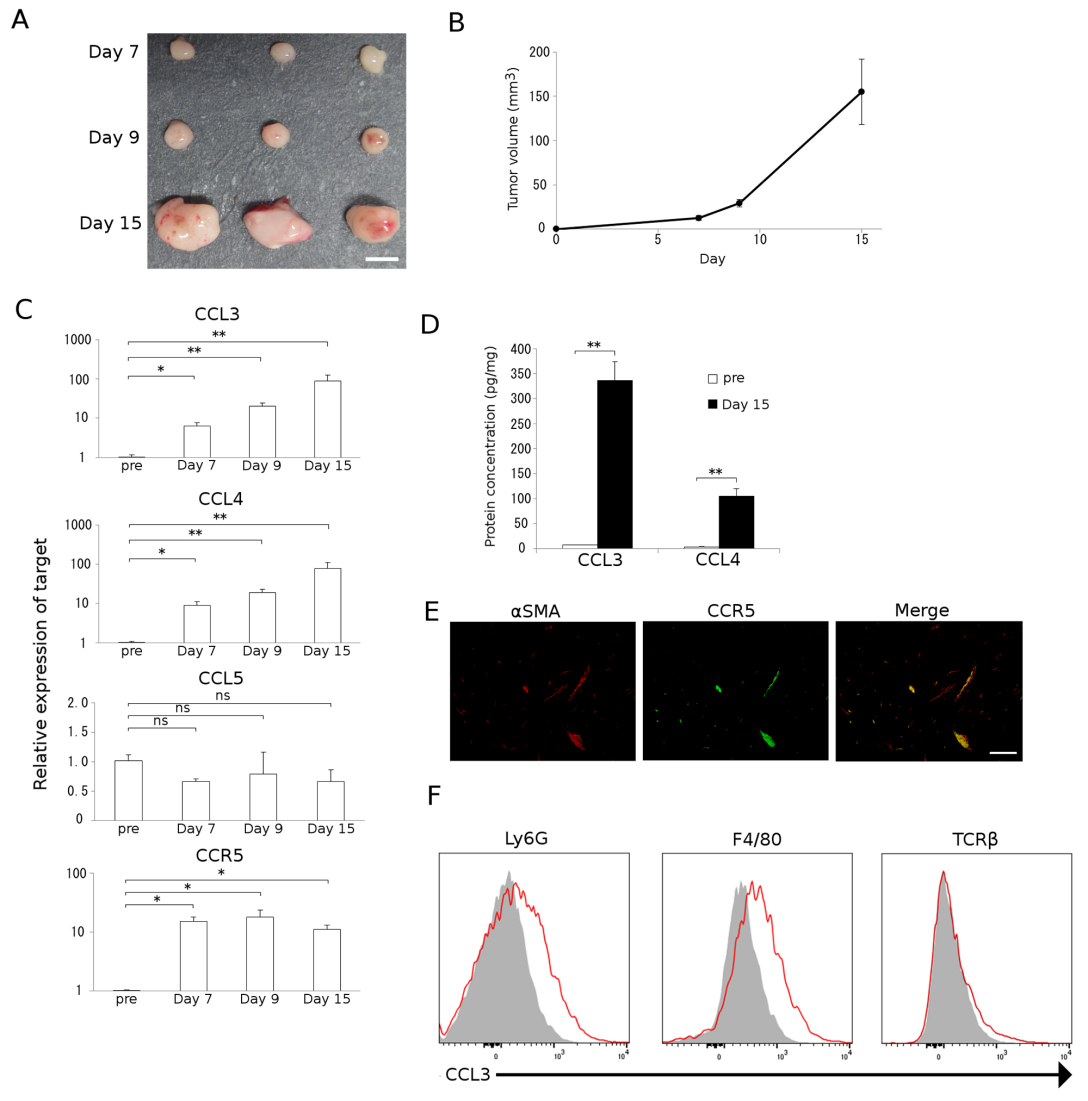
Colon carcinoma progression with CCL3-expressing resident stromal cell accumulation **A** and **B.** A mouse colon adenocarcinoma cell line, colon 26, was injected into the cecal wall of WT mice as described in Materials and Methods. Tumors were resected at the indicated time intervals from the injection. Representative macroscopic appearance of tumors is shown in A. Tumor volumes were determined at the indicated time intervals. Mean and SD were calculated and are shown in B (n = 8). **C.** Expression of *Ccr5* and its ligands in tumor tissues. Total RNAs were extracted from the tumor tissues obtained at the indicated time intervals and subjected to qRT-PCR to determine the mRNA expression of CCR5 and its ligands, CCL3, CCL4, and CCL5. Data represent the means and SEM (n = 5). Statistical significance was evaluated using one-way ANOVA, followed by Dunnett's test. *p<0.05; **p<0.01; ns, not significant. **D.** CCL3 and CCL4 protein expression in tumor tissues. Tumor tissues were obtained at the indicated time intervals and the levels of CCL3 and CCL4 protein in the tumor tissue extracts were quantified using ELISA. Data represent the means and SEM (n = 6). *p* values were calculated using Mann-Whitney's *U* test. **p<0.01. **E.** Double-color immunofluorescence analysis of CCR5-expressing cells using the combination of anti-CCR5 and anti-α-SMA antibodies. Representative results from 5 independent experiments are shown. The scale bar indicates 50 μm. **F.** Flow cytometry data of CCL3 expression by Ly6G-positive granulocytes, F4/80-positive macrophages, and TCRβ-positive T cells isolated from tumor tissues. Single cell suspensions were obtained from tumor tissues at 15 days after tumor inoculation and were subjected to flow cytometric analysis to determine the proportion of CCL3-expressing cells. Red lines and gray-filled histograms indicate the CCL3 expression and isotype-matched control, respectively. Representative results from three independent experiments are shown.

We previously demonstrated that tumor formation was significantly reduced in CCR5-deficient mice compared with WT mice when colon 26 cells were injected into the cecal wall [10]. These observations imply the possible involvement of the CCR5 axis in colon tumor progression. Hence, in the current study we inoculated colon 26 cells into the cecal wall of WT mice in order to delineate the molecular and cellular mechanisms underlying this phenomenon in more detail. Tumors were macroscopically evident 7 days after the injection and grew progressively (Figure 1A and 1B). We next determined the expression of CCR5 and its ligands in the tumor sites by quantitative reverse transcription-polymerase chain reaction (qRT-PCR) analysis. The expression of *Ccr5* and its ligands, *Ccl3* and *Ccl4*, but not *Ccl5*, were markedly increased in tumor sites 7 days after the injection (Figure 1C). Intratumoral CCL3 and CCL4 contents were also significantly increased at the protein level 15 days after the injection (Figure 1D). Furthermore, a double-color immunofluorescence analysis demonstrated that CCR5 was detected mainly in α-smooth muscle actin (SMA)-positive fibroblasts (Figure 1E). Flow cytometric analysis illustrated that CCL3 proteins were expressed in Ly6G-positive granulocytes and F4/80-positive macrophages but not in T cell receptor β chain (TCRβ)-positive T cells (Figure 1F).

### Maraviroc suppresses the tumor formation of a murine CRC cell line

**Figure 2:**
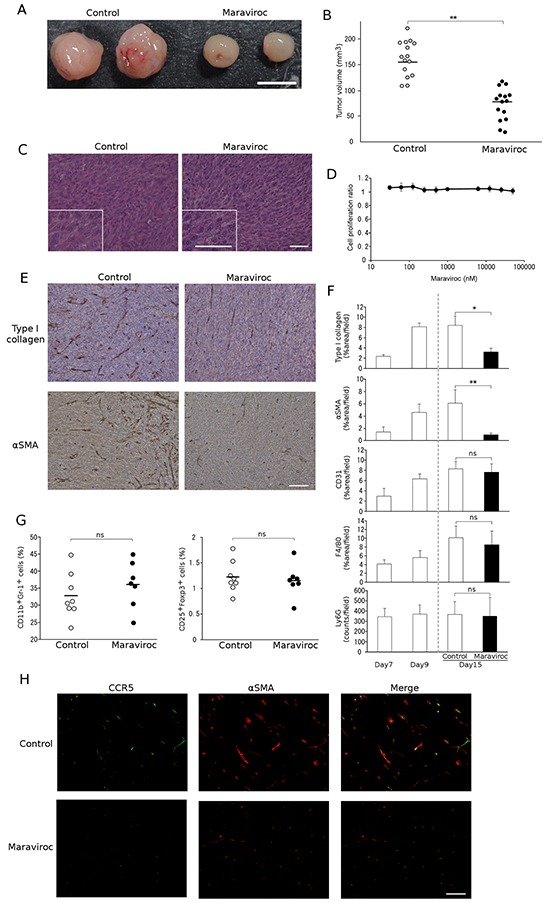
Effects of maraviroc on colon 26 cell tumor formation **A.** and **B.** Maraviroc was orally administered to mice that had received an injection of colon 26 cells into the cecal wall as described in Materials and Methods. Tumors were resected at day 15 after the injection. Representative macroscopic appearances of tumors are shown in A. Tumor volumes were determined and each symbol in B indicates the tumor volume of each mouse. The mean value was calculated for each group (n = 15) and is shown with a bar. *p* values were calculated using Mann-Whitney's *U* test. **p<0.01. **C.** Microscopic appearance of tumors. Tumors were resected at day 15 after the injection and were subjected to hematoxylin and eosin staining. Insets indicate high power magnification. Representative results from 5 individual animals are shown. The scale bar indicates 50 μm. **D.** Effects of maraviroc on the *in vitro* cell proliferation of colon 26 cells. *In vitro* cell proliferation was conducted in quadruplicate as described in Materials and Methods. The means and SD values were calculated and are as shown. **E.** and **F.** Tumor tissues were obtained at day 15 and subjected to immunohistochemical analysis using anti-Ly6G, anti-F4/80, anti-CD31, anti-type I collagen, or anti-α-SMA antibodies. Representative microscopic appearances of CAFs in tumor tissues are shown in E. The scale bar indicates 50 μm. Positive areas were determined as described in Materials and Methods. The means and SD values were calculated from 5 animals and are shown in F. Statistical significance was evaluated using one-way ANOVA, followed by Tukey-Kramer's posthoc test. *p<0.05; **p<0.01; ns, not significant. **G.** Immunosuppressive cell infiltration into inoculated tumors. Single cell suspensions were obtained from tumor tissues at 15 days after tumor inoculation and subjected to flow cytometric analysis to determine the populations of CD11b^+^Gr-1^+^ MDSCs and CD25^+^Foxp3^+^ Tregs among the CD45 cells. Each symbol indicates a separate mouse. The mean value was calculated for each group (control: n = 8, Maraviroc: n = 7) and is shown with a bar. *p* values were calculated using Mann-Whitney's *U* test. ns, not significant. **H.** Double-color immunofluorescence analysis of CCR5-expressing cells using anti-CCR5 and anti-α-SMA antibodies. Representative results from 5 independent experiments are shown. The scale bar indicates 50 μm.

The potential involvement of the CCR5 axis in colon tumor progression prompted us to investigate the effects of a CCR5 antagonist, maraviroc, on colon tumor progression induced by the injection of colon 26 cells into the cecal wall. Maraviroc treatment reduced tumor sizes by approximately half (Figure 2A and 2B). However, maraviroc treatment failed to induce massive necrosis and apoptosis in tumor cells (Figure 2C). Similar observations were obtained when maraviroc administration was instituted beginning at 8 days after the tumor injection (data not shown). Consistent with these findings, maraviroc exhibited few effects on the *in vitro* cell proliferation of colon 26 cells up to 100 μM (Figure 2D). These observations suggest that maraviroc might act on other types of cells present in tumor tissues rather than on the cancer cells themselves.

### Maraviroc suppresses fibroblast accumulation in tumors arising from the orthotopic injection of a murine CRC cell line

We next evaluated the effects of maraviroc on non-cancer cells present in tumor tissues, such as endothelial cells, fibroblasts, and leukocytes. Immunohistochemical analysis demonstrated that tumor tissues additionally contained CD31-positive endothelial cells and type I collagen- or α-SMA-positive fibroblasts. Furthermore, maraviroc significantly reduced type I collagen- and α-SMA-positive areas but not CD31-positive areas (Figure 2E and 2F). Infiltrating leukocytes were comprised mainly of Ly6G-positive granulocytes and F4/80-positive macrophages with a small number of CD3-positive lymphocytes (data not shown); however, maraviroc had few effects on these infiltrating leukocyte percentages (Figure 2F). Although CCR5 was detected in immunosuppressive cells [11, 12], maraviroc failed to change the intratumoral percentages of myeloid-derived suppressor cells (MDSCs) and regulatory T cells (Tregs), two major immunosuppressive cell types (Figure 2G). On the contrary, a double-color immunofluorescence analysis demonstrated that maraviroc reduced CCR5-expressing α-SMA-positive fibroblasts (Figure 2H). These observations imply that maraviroc reduced tumor formation mainly by acting on fibroblasts.

### Maraviroc suppresses the migration of fibroblasts expressing epidermal growth factor (EGF)

**Figure 3:**
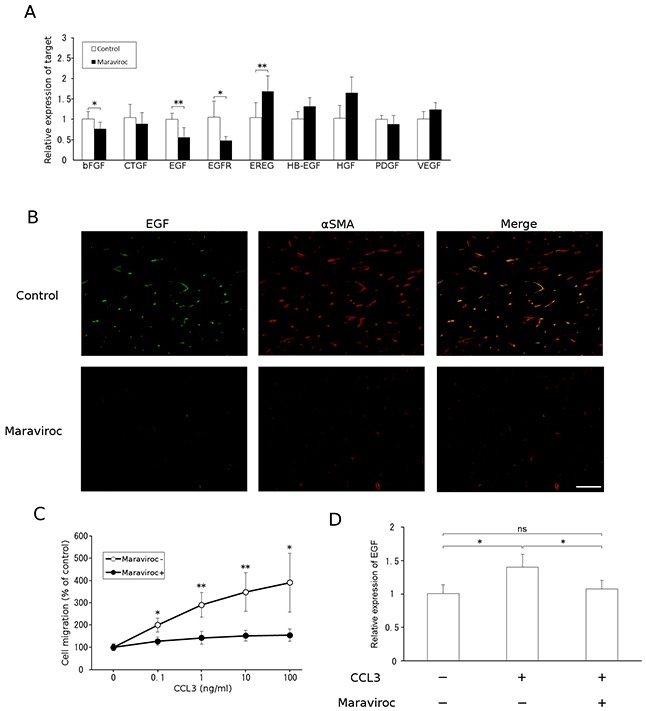
EGF expression by CAFs **A.** Total RNAs were extracted from tumor tissues obtained at day 15 after the injection and subjected to qRT-PCR analysis to determine the expression of growth factors. The means and SD values were calculated from 5 animals and are as shown. *p* values were calculated using Mann-Whitney's *U* test. *p<0.05; **p<0.01. **B.** Double-color immunofluorescence analysis of EGF-expressing cell was conducted with anti-EGF and anti-α-SMA antibodies. Representative results from 5 independent experiments are shown. The scale bar indicates 50 μm. **C.** Migration of fibroblasts in response to CCL3. Each value represents the means and SEM (n = 5). *p* values were calculated using Mann-Whitney's *U* test. *p<0.05; **p<0.01. **D.** Effects of CCL3 on fibroblast *EGF* expression. NIH3T3 cells were treated with CCL3 in the presence or absence of maraviroc, as described in Materials and Methods. Total RNAs were extracted to determine *Egf* mRNA levels using qRT-PCR. The means and SD values were calculated from 8 independent experiments and are as shown. Statistical significance was evaluated using one-way ANOVA followed by Tukey-Kramer's posthoc test. *p<0.05; ns, not significant.

Accumulating evidence indicates that CAFs are a rich source of various growth factors including hepatocyte growth factor (HGF), EGF, heparin-binding epidermal growth factor-like growth factor (HB-EGF), and epiregulin (EREG). Among these, only *Egf* mRNA expression was reduced by maraviroc treatment in tumor tissues (Figure 3A). In addition, a double-color immunofluorescence analysis revealed that EGF proteins were detected in α-SMA-positive cells and that maraviroc treatment reduced EGF-expressing and α-SMA-positive cells (Figure 3B). Furthermore, *in vitro* exposure to CCL3 enhanced cell migration and EGF expression in a mouse fibroblast cell line; this enhancement was abrogated by maraviroc treatment (Figure 3C and 3D). Although CCL3 marginally increased EGF expression, the effect of CCL3 on fibroblast migration was evident. These observations suggest that maraviroc primarily blocked the CCR5-induced intratumoral accumulation and subsequent activation of fibroblasts expressing EGF, an essential growth factor for CRC cells [13].

### Maraviroc suppresses the tumor formation of a human CRC cell line

**Figure 4:**
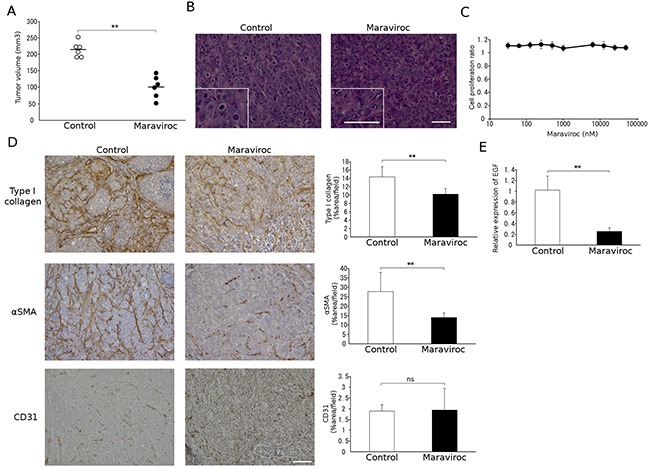
Effects of maraviroc on KM12C cell tumor formation **A.** and **B.** Maraviroc was administered orally to mice that had received an injection of KM12C cells into the cecal wall. Tumors were resected at day 28 after the injection to determine tumor volumes. Each symbol indicates the tumor volume of an animal and the means of each group (n = 6) is indicated with a bar. *p* values were calculated using Mann-Whitney's *U* test. **p<0.01. Representative microscopic appearance of hematoxylin and eosin-stained tumor tissues is shown in B; the insets indicate high power magnification. The scale bar indicates 50 μm. **C.** Effects of maraviroc on the *in vitro* cell proliferation of KM12C cells. *In vitro* cell proliferation was conducted in quadruplicate as described in Materials and Methods. The means and SD values were calculated and are as shown. **D.** Tumor tissues were obtained at day 28 after the injection and subjected to immunohistochemical analysis using anti-CD31, anti-type I collagen, or anti-α-SMA antibodies. Positive areas were determined. The means and SD values were calculated from 5 animals and are as shown. *p* values were calculated using Mann-Whitney's *U* test. **p<0.01; ns, not significant. **E.** Total RNAs were extracted from tumor tissues obtained at day 28 after the injection and subjected to qRT-PCR analysis to detect *Egf* mRNA expression. The means and SD values were calculated from 5 animals and are as shown. The data were analyzed statistically using Mann-Whitney's *U* test. **p<0.01.

To generalize the results obtained in the murine model, we next examined the effects of maraviroc on tumor formation induced by the injection of a human CRC cell line, KM12C, into the cecal wall. As for colon 26 cells, maraviroc treatment reduced xenotransplant tumor formation without inducing apparent necrosis in the tumor cells (Figure 4A and 4B). Consistent with these findings, maraviroc failed to inhibit the *in vitro* cell proliferation of KM12C cells up to 100 μM (Figure 4C), similarly to its observed lack of effect on colon 26 cells.

### Maraviroc suppresses fibroblast accumulation in tumors arising from the orthotopic injection of a human CRC cell line

We next examined the effects of maraviroc on the cell components in tumors arising from the injection of KM12C into the cecal wall. Maraviroc reduced intratumoral type I collagen- or α-SMA-positive fibroblast areas with few effects on CD31-positive areas (Figure 4D) or on Ly6G-positive granulocyte and F4/80-positive macrophage percentages in the tumor sites (data not shown). Furthermore, maraviroc treatment significantly attenuated mouse *Egf* mRNA expression (Figure 4E). Thus, maraviroc likely inhibits the tumor formation induced by the injection of a human CRC cell line into the cecal wall mainly by acting on CAFs.

## DISCUSSION

Fibroblasts are a major cell component of the tumor microenvironment in various types of solid tumors [7]. Under homeostatic conditions, fibroblasts can act as a sentinel cell to restrict pre-malignant cell growth [14]. In contrast, CAFs can also contribute to tumor progression by providing cancer cells with growth factors [8, 9] and pro-inflammatory tumor-promoting microenvironments [15], and by inducing neovascularization [16]. Furthermore, stromal markers are robustly associated with disease relapse in human CRC, which can arise from an increased frequency of tumor-initiating cells enhanced by CAFs [17]. Thus, drug dampening CAFs might be able to supplement currently used anti-cancer treatments. We previously unraveled the crucial role of the CCL3-CCR5 axis in CAF accumulation in colitis-associated carcinogenesis and tumor formation arising from injection of the mouse CRC cell line colon 26 either underneath the skin or into the cecal wall [10]. Hence, in the current study we investigated the therapeutic effects of a CCR5 antagonist, maraviroc, on preclinical models using a mouse or a human CRC cell lines and demonstrated that maraviroc markedly attenuated tumor formation as well as CAF accumulation with few effects on infiltrating leukocyte numbers.

The identification of CCR5 as a crucial co-receptor for human immunodeficiency virus (HIV) [18] promoted the development of a CCR5 antagonist to be applied to the treatment of HIV infection. Maraviroc, the only CCR5 antagonist currently approved in various countries [19], binds to a site that is distinct from the proposed recognition sites for chemokines and the HIV glycoprotein gp120 [20]. The maraviroc binding site consists of 16 amino acids, 15 of which are common between mouse and human CCR5 proteins. Furthermore, several independent groups have successfully used maraviroc to inhibit the mouse CCR5 axis [21–27]. These observations incited us to utilize maraviroc in these pre-clinical studies.

Initial immunofluorescence analysis detected CCR5 expression in a subpopulation of T lymphocytes associated with certain immune reactions, particularly Th1 type reactions [28]. In addition, the interaction between CCR5 and its ligand, CCL5, recruits Tregs [11] and tumor-associated macrophages [29, 30] as well as increases the generation of MDSCs [12]. Thus, CCR5 blockade can counteract these immune suppressive cells to enhance tumor immunity and to eventually reduce tumor growth. However, CCR5 expression was barely detected in infiltrating lymphocytes and macrophages in our model and maraviroc did not markedly change the macrophage, Treg, and MDSC numbers. Thus, it is unlikely that maraviroc acted by reducing the infiltrating immunosuppressive cells to retard tumor growth in this model.

Several independent groups have observed that CCR5 was expressed by some types of cancer cells including breast [31], prostate [32], and gastric cancer [33]. Furthermore, in these studies, maraviroc directly inhibited the *in vivo* invasiveness of these cancer cells and reduced subsequent tumor progression. In contrast, CCR5 blockade was reported to enhance the *in vivo* proliferation of breast cancer cells bearing wild-type p53 [34]. Thus, the functionality of the CCR5 expressed by cancer cells remains elusive. Nevertheless, the lack of CCR5 expression in colon 26 or KM12C cells argues against the direct actions of maraviroc on cancer cells in our present preclinical models.

Another potential mechanism for the observed maraviroc-mediated tumor reduction in our model is suggested by the results from Ohshio et al., who administered a clinically used anti-fibrotic agent, tranilast, to tumor-bearing mice [35]. Several lines of evidence indicate that tranilast mainly interferes with the transforming growth factor (TGF)-β-mediated signaling pathway to inhibit fibrosis [36]. Tranilast was shown to significantly reduce CAFs but had only marginal effects on *in vivo* tumor growth [35]. Conversely, maraviroc treatment attenuated tumor growth as well as CAF number. In addition, maraviroc has been found to be well tolerated by patients with acquired immune deficiency syndrome without increasing the incidence of severe adverse effects [37]. Thus, several groups have proposed the use of maraviroc for the treatment of other diseases wherein CCR5 was presumed to be implicated [19]. Given the crucial roles of the CCR5 axis in CAF accumulation and function, maraviroc application might therefore also be useful in the treatment of cancers with an abundant number of CAFs.

## MATERIALS AND METHODS

### Mice

Specific pathogen-free female 7-week-old BALB/c mice (wild-type; WT mice) and male 7-week-old BALB/c-nu mice (nude mice) were purchased from Charles River Japan (Yokohama, Japan). All mice were maintained under specific pathogen-free conditions. All the animal experiments in this study were approved by the Committee on Animal Experimentation of Kanazawa University and complied with the Guideline for the Care and Use of Laboratory Animals of Kanazawa University.

### Reagents and antibodies

Maraviroc was obtained from Sigma-Aldrich (St. Louis, MO, USA) (for *in vitro* experiments) or GlaxoSmithKline (Brentford, UK) (for *in vivo* experiments). Mouse CCL3 was obtained from Peprotech (Rocky Hill, NJ, USA). The following antibodies were used as the primary antibodies for immunohistochemical analyses: goat anti-CCR5 antibody (Santa Cruz Biotechnology, Dallas, TX, USA), rat anti-Ly6G antibody (BD Biosciences, San Jose, CA, USA), rat anti-F4/80 antibody (Serotec, Kidlington, UK), mouse anti-α-SMA antibody (Dako, Glostrup, Denmark), rabbit anti-type I collagen antibody, rabbit anti-CD31 antibody and rabbit anti-EGF antibody (Abcam, Cambridge, UK). The following rat anti-mouse antibodies were used as the primary antibodies for the flow cytometric analysis: anti-CD11b antibody (BD Biosciences), anti-CD25 antibody (BioLegend, San Diego, CA, USA), anti-CD45 antibody (eBioscience, San Diego, CA, USA), anti-F4/80 antibody (eBioscience), anti-Foxp3 antibody (eBioscience), anti-Ly6G antibody (Gr-1) (Tonbo Biosciences, San Diego, CA, USA), anti-MIP-1α antibody (R&D Systems, Minneapolis, MN, USA), anti-CCR5 antibody (BioLegend), and isotype-matched control IgGs for individual rat antibodies (BD Biosciences).

### Cell lines

A murine CRC cell line, colon 26 (CRL-2638; American Type Culture Collection (ATCC), Manassas, VA, USA) and a human CRC cell line, KM12C (a gift from Dr. I. J. Fidler, M.D. Anderson Cancer Center) were maintained in RPMI 1640 medium supplemented with 10% fetal bovine serum (FBS). A mouse fibroblast cell line, NIH3T3 (CRL-6361; ATCC) was maintained in Dulbecco's modified Eagle's medium supplemented with 10% FBS. These cells were cultured at 37°C under 5% CO_2_.

### *In vitro* cell proliferation of CRC cell lines

Cell suspensions (2 × 10^3^ cells /100 μl) were added to each well of 96-multi-well plates and incubated at 37°C for 24 h. The cells were then treated with the indicated concentrations of maraviroc for 72 h. The cell viability was determined using the cell counting kit-8 (Dojindo Co. Ltd., Kumamoto, Japan), which is based on the determination of the extracellular WST-8 reduction by NADH produced in the mitochondria resulting in soluble formazan. The ratios of cell proliferation were determined by comparison to the number of untreated cells.

### *In vitro* experiments using NIH3T3 cells

Sub-confluent NIH3T3 cells were seeded at a cell density of 3 × 10^5^ cells/ml in each well of a 24-multi-well plate. After 24 h later, the cells were treated with 100 nM maraviroc for 1 h. Thereafter, mouse CCL3 was added to each well at 100 ng/ml. The cells were harvested to extract total RNA at 6 h after the addition of CCL3 as described below. The resultant total RNA was subjected to qRT-PCR. For *in vitro* cell migration analysis, the indicated concentrations of mouse CCL3 and 50,000 cells were placed in the lower and upper chambers of 24-well trans-well chambers (8 μM pore size; Costar, Cambridge, MA, USA), respectively, in the presence or absence of 100 nM maraviroc, and were incubated at 37°C for 24 h in 5% CO_2_. After gently removing the upper chambers, the numbers of cells that had passed to the lower chambers were counted.

### Orthotopic tumor formation

Subconfluent colon 26 and KM12C cells were collected and resuspended in Hank's balanced salt solution at a cell density of 1.25 × 10^6^ and 2.5 × 10^7^ cells/ml, respectively. The cell viability was confirmed to be always >95% using a trypan blue exclusion test. Prior to surgery, mice were anesthetized by an intraperitoneal injection of reagent containing medetomidine (0.3 mg/kg), midazolam (4 mg/kg), and butorphanol (5 mg/kg). A small incision was made along the midline of the abdomen and the cecum was exteriorized. Then, 40 μl cell suspensions of colon 26 and KM12C cells were injected into the cecal wall of WT and nude mice, respectively, using a 30-gauge needle. After surgery, the abdominal muscle wall was closed using surgical silk sutures. Mice also received an intraperitoneal injection of the anti-medetomidine inhibitor, atipamezole (0.3 mg/kg). From 2 days after the tumor injection, mice were administered maraviroc at a dose of 30 mg/kg every 2 days. At the indicated time intervals from the inoculation, cecum was removed for the determination of tumor volume, immunohistochemical analysis, and total RNA extraction followed by qRT-PCR. Tumor volumes were calculated according to the following equation: π × a × b × c/6, where a, b, and c indicate the width, depth, and height of the tumor, respectively.

### Histological and immunohistochemical analysis

Resected tumor tissues were fixed in 10% formaldehyde and embedded in paraffin. The sections were cut at 4-μm thickness and stained with hematoxylin and eosin solution. In parallel, 5-μm-thick sections were generated for immunohistochemical analysis. For antigen retrieval of paraffin sections, the deparaffinized slides were either autoclaved in 10 μM citrate buffer (pH 6.0) for 15 min at 121°C (for staining with anti-type I collagen, anti-CD31 or anti-α-SMA), treated with 0.1% proteinase K solution for 15 min at 37°C (for staining with anti-EGF or anti-CCR5), or treated with 0.1% trypsin solution for 15 min at 37°C (for staining with anti-F4/80 or anti-Ly6G). Endogenous peroxidase activity was blocked using 0.3% H_2_O_2_ for 15 min, followed by incubation with Blocking One Histo (Dako Japan, Kyoto) for 15 min. The sections were further incubated with the optimal dilution of the antibodies overnight in a humidified box at 4°C. The resultant immune complexes were detected by the ABC Elite kit (Vector Laboratories, Burlingame, CA, USA) and peroxidase substrate 3-3′-diaminobenzidine kit (Vector), according to the manufacturer's instructions. Positive signals or areas were measured on 5 randomly chosen visual fields at ×100 magnification by means of Image J (National Institutes of Health, Bethesda, MD, USA). For double-color immunofluorescence analysis, Alexa Fluor 594 donkey anti-mouse, Alexa Fluor 488 donkey anti-goat antibody, or Alexa Fluor 488 donkey anti-rabbit antibody (Invitrogen, Carlsbad, CA, USA) were used as secondary antibodies. Immunofluorescence was visualized on a KEYENCE BZ-X710 fluorescence microscope (Osaka, Japan).

### qRT-PCR analysis

Total RNAs were extracted from the cell lines or tumors using the RNeasy Mini Kit (Qiagen, Venlo, The Netherlands). After treatment with RNase-free DNase I (Qiagen), 2 μg total RNA was reverse-transcribed using the SuperScript III First-Strand Synthesis System (Invitrogen) to obtain cDNA. qPCR was performed on the obtained cDNA using the StepOne real-time PCR system (Applied Biosystems, Foster City, CA, USA) using the Quantifast SYBR Green PCR Master mix (Applied Biosystems) and the primers listed in Table 1. Expression levels of the target genes were analyzed using the ΔΔCT comparative threshold method. The glyceraldehyde-3-phosphate dehydrogenase (*Gapdh*) gene was used as an internal control.

**Table 1:** Nucleotide sequence of the primers used for qRT-PCR

Gene name	Species	Size	Forward primer (5′-3′)	Reverse primer (5′-3′)
*Gapdh*	mouse	55	catggccttccgtgttccta	gcggcacgtcagatcca
*Ccr5*	mouse	61	catccgttccccctacaaga	ggaactgacccttgaaaatcca
*Ccl3*	mouse	57	gctgacaagctcaccctctgt	ggcagtggtggagaccttca
*Ccl4*	mouse	57	cagcaccaatgggctctga	gccgggaggtgtaagagaaac
*Ccl5*	mouse	80	tccaatcttgcagtcgtgtttg	tctgggttggcacacacttg
*Fgfb*	mouse	127	gacccacacgtcaaactacaactc	ctgtaacacacttagaagccagcag
*Ctgf*	mouse	232	ggtgagtccttccaaagcag	ggccaaatgtgtcttccagt
*Egf*	mouse	111	tgcctcagaaggagtgggtta	gtgttccaagcgttcctgaga
*Egfr*	mouse	204	gaactgggcttagggaactgc	cattgggacagcttggatcac
*Ereg*	mouse	166	taccgccttagttcagatgg	acatcgcagaccagtgtagc
*Hb-egf*	mouse	111	gcaaatgcctccctggttac	ctacagccaccacagccaaga
*Hgf*	mouse	57	tcggataggagccacaagga	ccgaggccagctgcaat
*Pdgf*	mouse	154	gcaccaacgccaacttcct	atgggcttctttcgcacaat
*Vegf*	mouse	234	ctactgccgtccgattgaga	catctgctgtgctgtaggaag

### Flow cytometry

Tumor tissues were obtained from the cecum. After removing the epithelial cells, the tissues were further cut into small pieces and incubated with RPMI 1640 containing 1 mg/ml collagenase type I and 40 μg/ml DNase I for 20 min at 37°C with shaking for 180 rpm. The resulting digestion mixtures were filtered through a 100-μm cell strainer (Corning, Cambridge, MA, USA) to achieve single cell suspensions. Isolated single cell suspensions were stained with various combinations of fluorescent dye-conjugated antibodies in MACS buffer. For intracellular MIP-1α/CCL3 staining, isolated single cell suspensions were incubated in serum-free RPMI 1640 medium supplemented with 0.1% GolgiStop reagent (BD) for 4 h. Subsequently, intracellular CCL3 was stained with PE-conjugated anti-CCL3 antibody assisted by an Intracellular Cytokine Staining Starter kit (BD). Intracellular staining for Foxp3 was performed using a Foxp3 Staining kit (BD). Expression of each molecule was determined using FACSCanto II (BD) and analyzed with FlowJo software (Tree Star, Ashland, OR, USA).

### Enzyme-linked immunosorbent assay (ELISA)

The tissues collected at the indicated time intervals were homogenized with a lysis buffer (20 mM Tris-HCl, pH 7.6; 150 mM NaCl; 1% Triton; and 1 mM EDTA) containing Complete Protease Inhibitor Cocktail (Roche-Diagnostics, Roswell, GA, USA). Homogenates were centrifuged to obtain protein lysates. After the determination of protein concentration using the BCA Protein Assay kit (Pierce, Rockford, IL, USA), CCL3 and CCL4 levels were measured in the protein lysates using the specific ELISA kits (R&D Systems), according to the manufacturer's instructions.

### Statistical analysis

The data were statistically analyzed using the methods indicated in each figure legend. *p* values less than 0.05 were considered statistically significant.
